# Unbiased weighted variance and skewness estimators for overlapping returns

**DOI:** 10.1186/s41937-018-0023-1

**Published:** 2018-11-17

**Authors:** Stephen Taylor, Ming Fang

**Affiliations:** Martin Tuchman School of Management at the New Jersey Institute of Technology, Newark, NJ USA

**Keywords:** Overlapping returns, Variance and skewness estimation, Asset returns, Weighted estimators

## Abstract

This article develops unbiased weighted variance and skewness estimators for overlapping return distributions. These estimators extend the variance estimation methods constructed in Bod et. al. (Applied Financial Economics 12:155-158, 2002) and Lo and MacKinlay (Review of Financial Studies 1:41-66, 1988). In addition, they may be used in overlapping return variance or skewness ratio tests as in Charles and Darné (Journal of Economic Surveys 3:503-527, 2009) and Wong (Cardiff Economics Working Papers, 2016). An example using synthetic overlapping returns from a model fit to data from the SPY S&P 500 exchange traded fund is given in order to demonstrate under which circumstances the unbiased correction becomes significant in skewness estimation. Finally, we compare the effect of the HAC weighting schemes of Andrews (Econometrica 53:817-858, 1991) as a function of sample size and overlapping return window length.

## Introduction

Overlapping returns are used in many contexts in the finance and econometrics literature. Applications include variance ratio tests, regression parameter error estimation, and alternative resampling methods. Standard statistical inference and estimation techniques applied to overlapping return financial time series are typically biased. In addition, for such series, recent data is regularly viewed as more relevant than past information, which has resulted in the creation of weighted generalizations of estimation methodologies. This motivates the development of unbiased analogues of such estimators which we explore in the cases of the variance and skewness statistics. Our central aim is to construct unbiased weighted variance and skewness estimators for overlapping return distributions.

Several estimation procedures and hypothesis testing frameworks have been improved through the utilization of overlapping returns. In financial overlapping return applications, Lo and MacKinlay (1988) and Hansen and Hodrick (1980) demonstrate how overlapping returns may be used to increase the efficiency of statistics used in variance ratio tests. Dunis and Keller (1995) developed a panel regression method based on overlapping returns, and Müller (1993) concludes utilizing overlapping returns in most applications will result in an overall increase in estimation precision of statistics that are a function of the overlapping returns when compared with their analogues for simple returns. In Jackwerth (2000), the author discovered that the overlapping return distribution for the S&P 500 is left-skewed and examined differences between risk neutral and realized distributions between overlapping and non-overlapping returns of the S&P 500 index and observed how associated risk aversion functions changed dramatically around the 1987 stock market crash. Wong (2016) develops skewness and kurtosis ratio tests for overlapping returns. The new weighted unbiased skewness estimator constructed below may be used as an input into any of these applications.

The idea of assigning greater weight to recent data and less weight to past data has been discussed in a number of econometric and financial studies. Past economic data may have little impact or be entirely irrelevant for present projections. In addition, by placing additional weight on recent data, associated estimation procedures tend to react more strongly to structural changes in the underlying assumption about the distribution the sample is drawn from than their uniformly weighted counterparts. For example, Tsokos (2010) shows that under nonstationary economic realization, weighted moving average models perform significantly better than the classical ARIMA model in forecasting stock prices. Andrews (1991) develops weighting schemes used in the estimation of covariance matrices assuming the underlying time series exhibits nontrivial autocorrelation and heteroskedasticity which we will utilize below. Weighted estimators are routinely used in practice as well. In particular, in Longerstaey and Spencer (1996), it is demonstrated that exponentially weighted moving average estimators incorporate external shocks more readily than equally weighted moving averages, thus providing a more realistic measure of current volatility.

Volatility and skewness estimation of financial return distributions has been the subject of a number of articles. Early examples include using maximum likelihood estimation to fit a model distribution to observed data and computing the associated model statistics in Fama (1965) and Mandelbrot (1963). More recent work has focused on the estimation of stochastic volatility models in Broto (2004). Time series techniques have also been widely applied to this task, c.f. (Tsay 2010). Measuring the asymmetry of financial return distributions has also been the central theme of many references. Grigoletto and Lisi (2006) and Wen and Yang (2009) find persistent non-trivial skewness is present in the simple daily return distributions of nearly every major international equity index. Xu (2007) shows that equity return distribution skewness is positively correlated with simultaneous returns and negatively correlated with lagged returns.

When working with overlapping returns, especially when encountering small sample sizes, bias effects from standard estimators, such as the sample variance, become important. In Lo and MacKinlay (1988), the authors provide a consistent but biased overlapping return variance estimator that has been used in several subsequent references, including Liu and He (1991), Fong et al. (1997), and Amélie and Olivier (2009). This estimator was improved in Bod et al. (2002) where the authors constructed an unbiased variance estimator for unweighted overlapping returns. Kluitman and Franses (2002) extended this work to develop an estimator that includes the case where the returns have nontrivial autocorrelation. Our main contribution is to extend these results by developing weighted unbiased variance and skewness estimators for overlapping return time series.

This article is organized as follows. We first fix notation and then derive an unbiased weighted estimator for the variance of a time series of overlapping returns. We give reduced expressions for this estimator in the cases of uniform and exponential weights. Next, we construct a similar weighted unbiased estimator for the skewness of an overlapping return distribution. We then demonstrate the difference between a normalized version of the skewness estimator and the standard normalized sample skewness in a simulation which models the overlapping return distribution of the S&P 500 index, and then summarize our results. We finally compare the estimation of the weighted volatility and skewness of the overlapping return distribution of the S&P 500 index for various weighting schemes, sample sizes, and overlapping lengths and conclude with potential additional questions to explore.

## Methodology

We begin by establishing notation. Given integers *n,q*>0 with *q*<*n*, let *p*_*t*_>0 for *t*=0,…,*n*+*q*−1 denote an asset price time series by *p*_*t*_ and let *r*_*t*_=*p*_*t*_/*p*_*t*−1_−1 be the associated simple returns where *t*=1,…,*n*+*q*−1. Following Bod et al. (2002) and Lo and MacKinlay (1988), we assume that *r*_*t*_ have zero mean, $\mathbb {E}[\!r_{t}]=0$, covariance *E*[ *r*_*t*_*r*_*s*_]=0 for any *t*>*s*, and equal finite variance Var(*r*_*t*_)=*σ*^2^<*∞*. We note that in Lo and MacKinlay (1988) and subsequent references, the authors show that these assumptions, referred to as the random walk version of the martingale hypothesis, do not hold for a variety of financial time series. This is achieved by assuming a null hypothesis that they hold and then demonstrating how variance ratios of overlapping returns may be used to reject this assertion. In this spirit, we proceed by defining the *q*-period overlapping returns *y*_*t*_ associated with *r*_*s*_ by

1 $$ y_{t} = \sum_{s=t}^{t+q-1}{r_{s}},\quad\text{for}\quad t = 1,\ldots,n.  $$

We construct weighted unbiased estimators of the variance and skewness of *y*_*t*_ and pair a weight *w*_*t*_ with each *y*_*t*_ such that *w*_*t*_>0 and $\sum _{t=1}^{n}w_{t}= 1$. Let $W^{ts}=\sum _{k=t}^{s}w_{k}$ be the sum of the *t*-th through *s*-th weight, and note *W*^1*n*^=1.

We first derive an unbiased weighted overlapping return variance estimator $\hat {\sigma }_{y}^{2}$. We seek an estimator of the form

2 $$ \hat{\sigma}^{2}_{y} = C_{1}(n,q,w)^{-1}\sum_{t=1}^{n} w_{t} \left(y_{t}-\bar{y}^{w}\right)^{2}, \quad \bar{y}^{w} \equiv \sum_{t=1}^{n} w_{t} y_{t},  $$

where we find *C*_1_ such that $\hat {\sigma }_{y}^{2}$ is an unbiased estimator of *qσ*^2^. Note that since *r*_*i*_ are independent, the true variance of each *y*_*t*_ is Var(*y*_*t*_)=*qσ*^2^ so that $\hat {\sigma }^{2}$ is an unbiased estimator of the variance of the random variable from which *y*_*t*_ are sampled from if

3 $$ \mathbb{E}\left[\hat{\sigma}^{2}_{y}\right] = q\sigma^{2}.  $$

This may be viewed as a constraint that defines the constant *C*_1_(*n,q,w*) which we determine by computing

4 $$ \begin{aligned} &\mathbb{E}\left[\sum_{t} w_{t}\left(y_{t}-\bar{y}^{w}\right)^{2}\right] =\\[-4pt]&\qquad\qquad\qquad \sum_{t} w_{t}\left(\mathbb{E}y_{t}^{2}-2\mathbb{E}\left(y_{t}\bar{y}^{w}\right)+ \mathbb{E}\left[\left(\bar{y}^{w}\right)^{2}\right]\right), \end{aligned}  $$

and noting that

5 $$ \sum_{t} w_{t}\mathbb{E}\left(y_{t}\bar{y}^{w}\right) =\mathbb{E}\left(\bar{y}^{w} \sum_{t} w_{t}y_{t} \right) =\mathbb{E}\left[\left(\bar{y}^{w}\right)^{2}\right],  $$

yields

6 $$ \sum_{t} w_{t}\left(-2\mathbb{E}\left(y_{t}\bar{y}^{w}\right)+\mathbb{E}\left[\left(\bar{y}^{w}\right)^{2}\right]\right) =-\mathbb{E}\left[\left(\bar{y}^{w}\right)^{2}\right].  $$

Combining the above, we find

7 $$ \begin{aligned} \mathbb{E}\left[\sum_{t} w_{t}\left(y_{t}-\bar{y}^{w}\right)^{2}\right] &= \sum_{t} w_{t}\mathbb{E}y_{t}^{2} - \mathbb{E}\left[\left(\bar{y}^{w}\right)^{2}\right] \\&\quad= q\sigma^{2} - \text{Var}\left(\bar{y}^{w}\right), \end{aligned}  $$

where we note that $\mathbb {E}(y_{t})=\mathbb {E}\left (\bar {y}^{w}\right)=0$, and the last equality follows from $\mathbb {E}\left (y_{t}^{2}\right)=\text {Var}(y_{t})=q\sigma ^{2}$ as well as $\text {Var}\left (\bar {y}^{w}\right)=\mathbb {E}\left [\left (\bar {y}^{w}\right)^{2}\right ]-\mathbb {E}\left [\bar {y}^{w}\right ]^{2}=\mathbb {E}\left [\left (\bar {y}^{w}\right)^{2}\right ]$.

In order to compute the variance term in the previous equation, we decompose the weighted average of *y*_*t*_ as

8 $$ \bar{y}^{w} = \sum_{t=1}^{q-1}W^{1t}r_{t} + \sum_{t=q}^{n} W^{(t-q+1)t}r_{t} + \sum_{t=n+1}^{n+q-1} W^{(t-q+1)n}r_{t}.  $$

One may arrive at this decomposition by viewing the individual return terms of the sum $\bar {y}^{w} = \sum _{t} w_{t}y_{t}$ as a table with values *w*_*t*_*r*_*s*_ whose first row has elements, *w*_1_*r*_1_,*w*_1_*r*_2_,…,*w*_1_*r*_*q*_ and final row is given by *w*_*n*_*r*_*n*_,*w*_*n*_*r*_*n*+1_,…,*w*_*n*_*r*_*n*+*q*−1_. Note that $\bar {y}^{w}$ is equivalent to the sum of all values in this table. The first term in this decomposition corresponds to grouping all elements of this table above the diagonal whose edge is formed by the *w*_1_*r*_*q*_ and *w*_*q*_*r*_*q*_ entries and factoring our common returns multiplied into varying weights. The final term can be arrived at by aggregating all terms below the diagonal formed by the *w*_*n*−*q*+2_*r*_*n*+1_ and *w*_*n*_*r*_*n*+1_ entries. The middle sum is computed by combining the remaining terms in the table.

Since each individual sum is composed of returns that are independent of all the returns in the other two sums, we find that

9 $$ \begin{aligned} \text{Var}\left(\bar{y}^{w}\right) =\sigma^{2}\left[\sum_{t=1}^{q-1}\left(W^{1t}\right)^{2} + \sum_{t=q}^{n}\left(W^{(t-q+1)t}\right)^{2}+\sum_{t=n+1}^{n+q-1}\left(W^{(t-q+1)n}\right)^{2}\right]. \end{aligned}  $$

Solving for the unbiased constant from Eqs. (7) and (9), we arrive at

10 $$ \begin{aligned} C_{1}(n,q,w)& = \frac{1}{q\sigma^{2}}\mathbb{E}\left[\sum w_{t}\left(y_{t}-\bar{y}^{w}\right)^{2}\right] \\&\,=\,1\,-\,\frac{1}{q} \left[\sum_{t=1}^{q-1}\!\left(W^{1t}\right)^{2} \,+\,\sum_{t=q}^{n}\!\left(W^{(t-q+1)t}\right)^{2}\,+\,\sum_{t=n+1}^{n+q-1}\!\left(W^{(t-q+1)n}\right)^{2}\!\right]. \end{aligned}  $$

We note that in the case of uniform weights *w*_*t*_=1/*n*, this result reduces to that of Bod et. al. (2002), where $C_{B}^{-1}=nC_{1}(n,q,w)$. Secondly, in the case of exponential weights *w*_*s*_=*α*^*n*−*s*^/*C* with *C*=(*α*^*n*^−1)/(*α*−1), one can show

11 $$ {} C_{1}(n,q,w) \,=\, \frac{2\alpha}{q}\frac{\alpha^{q}-\alpha^{2n-q}-1+\alpha^{2n}- q\alpha^{n-1}\left(\alpha^{2}-1\right)}{\left(\alpha^{2}-1\right)(\alpha^{n}-1)^{2}}.  $$

We now derive an unbiased skewness estimator in a similar manner. Assume that $\mathbb {E}\left (r_{t}^{3}\right)=\gamma $, so that $\mathbb {E}\left (y_{t}^{3}\right)=q\gamma $, and consider an estimator of the skewness of the overlapping return distribution of the form

12 $$ \hat{\gamma}_{y} = C_{2}(n,q,w)^{-1}\sum_{t=1}^{n} w_{t} \left(y_{t} - \bar{y}^{w}\right)^{3}.  $$

We would like to construct an unbiased estimator of *qγ* which requires

13 $$ \mathbb{E}\left[\hat{\gamma}_{y}\right]=q\gamma.  $$

To determine *C*_2_, we compute

14 $$ {{} \begin{aligned} \mathbb{E}\left[\sum_{t=1}^{n} w_{t}\left(y_{t}-\bar{y}^{w}\right)^{3}\right] &= \sum_{t=1}^{n} w_{t} \mathbb{E}\Big[y_{t}^{3} -3y_{t}^{2}\bar{y}^{w} +\\ &\quad\quad\quad\quad\quad\left. 3y_{t}\left(\bar{y}^{w}\right)^{2}-\left(\bar{y}^{w}\right)^{3}\right]. \end{aligned}}  $$

Decomposing *y*_*t*_ into independent *r*_*t*_ sums allows one to calculate the first term with

15 $$ {\begin{aligned} \mathbb{E}\left(y_{t}^{3}\right) &= \mathbb{E}\left[\sum_{s=t}^{t+q-1}r_{s}^{3} +3\sum_{s\neq k} r_{s}^{2} r_{k} + \sum_{s\neq k \neq l} r_{s}r_{k}r_{l}\right] \\&= \sum_{s=t}^{t+q-1}\mathbb{E}\left(r_{s}^{3}\right) = q\gamma. \end{aligned}}  $$

For the second term, we note that

16 $$ \mathbb{E}\left[y_{t}^{2}\bar{y}^{w}\right] = \sum_{s} w_{s}\mathbb{E}\left(y_{t}^{2}y_{s}\right).  $$

Computing the expectation, we find,

17 $$ \begin{aligned} \mathbb{E}\left[y_{t}^{2}y_{s}\right] &= \mathbb{E}\left[\sum_{k=s}^{s+q-1}r_{k} \left(\sum_{l=t}^{t+q-1}r_{l}\right)^{2} \right] \\&=\mathbb{E}\left[\sum_{k=s}^{s+q-1}r_{k} \left(\sum_{l=t}^{t+q-1}r_{l}^{2} + \sum_{p\neq m}^{t+q-1}r_{m}r_{p}\right) \right] \end{aligned}  $$

18 $$ = \mathbb{E}\left[ \sum_{k=t}^{t+q-1}\left(r_{s}+\cdots+r_{s+q-1}\right)r_{k}^{2}\right].  $$

Note that only terms of the form $r_{t}^{3}$ are non-trivial in expectation. Splitting into two cases, we have

19 $$ \mathbb{E}\left[y_{t}^{2}y_{s}\right] = \left\{ \begin{array}{ll} (s-t+q)\gamma & s \leq t \leq s + q-1 \\ (t-s+q)\gamma & t \leq s \leq t + q-1 \\ \end{array}\right.  $$

20 $$ \hspace{35pt} = (q-|t-s|)\gamma,\quad 0\leq |t-s|\leq q-1.   $$

Combining Eqs. (16) and (20), we find that

21 $$ \sum_{t=1}^{n} w_{t}\mathbb{E}\left[y_{t}^{2}\bar{y}^{w}\right] = \gamma\sum_{t,s=1}^{n} w_{t}w_{s}(q-|t-s|)1_{\{|t-s|\in[0,q-1]\}}.  $$

For the final two terms, note

22 $$ \begin{aligned} \mathbb{E}\left(y_{t}\left(\bar{y}^{w}\right)^{2}\right) &= \sum_{s,k}w_{s}w_{k}\mathbb{E}(y_{t}y_{s}y_{k}), \quad \mathbb{E}\left(\bar{y}^{w}\right)^{3}\\&=\sum_{\text{\textit{t,s,k}}}w_{t}w_{s}w_{k}\mathbb{E}(y_{t}y_{s}y_{k}), \end{aligned}  $$

so we may simplify

23 $$ \sum_{t=1}^{n} w_{t}\left[3y_{t}\left(\bar{y}^{w}\right)^{2}-\left(\bar{y}^{w}\right)^{3}\right] = 2\mathbb{E}\left[\left(\bar{y}^{w}\right)^{3}\right].  $$

Using the decomposition in Eq. (14), by independence of the terms

24 $$ \begin{aligned} \mathbb{E}\left[\left(\bar{y}^{w}\right)^{3}\right] &= \gamma \left[\sum_{t=1}^{q-1}\left(W^{1t}\right)^{3} + \sum_{t=q}^{n} \left(W^{(t-q+1)t}\right)^{3} \right. \\[-4pt] & \quad \left.+\sum_{t=n+1}^{n+q-1}\left(W^{(t-q+1)n}\right)^{3}\right]. \end{aligned}  $$

Combining results from Eqs. (15), (21), and (24), we arrive at an expression for *C*_2_ given by

25 $$ \begin{aligned} C_{2}(n,q,w) &= 1 - \frac{3}{q}\sum_{\substack{t,s=1\\ |t-s|<q}}^{n} w_{t}w_{s}(q-|t-s|) \\ &\quad + \frac{2}{q} \left[\sum_{t=1}^{q-1}\left(W^{1t}\right)^{3} \!\,+\,\! \sum_{t=q}^{n} \left(W^{(t-q+1)t}\right)^{3} \,+\,\sum_{t=n+1}^{n+q-1}\left(\!W^{(t-q+1)n}\right)^{3}\!\right]\!. \end{aligned}  $$

In the case of uniform weights *w*_*t*_=1/*n*, the terms in this expression simplify to

26 $$ \begin{aligned} \sum_{\substack{t,s=1\\ |t-s|<q}}^{n} w_{t}w_{s}(q-|t-s|) &= \frac{q}{n}+\frac{2}{n^{2}}\sum_{t=1}^{q-1}(n-t)(q-t)\\ &=\frac{q}{3n^{2}}\left(1+3nq-q^{2}\right) \end{aligned}  $$

27 $$ \begin{aligned} \sum_{t=1}^{q-1}\left(W^{1t}\right)^{3} &+ \sum_{t=q}^{n} \left(W^{(t-q+1)t}\right)^{3} +\sum_{t=n+1}^{n+q-1}\left(W^{(t-q+1)n}\right)^{3} \\&\quad= \frac{1}{n^{3}}\left[\sum_{t=1}^{q-1}t^{3} \,+\, \sum_{t=q}^{n} q^{3} \,+\, \sum_{t=n+1}^{n+q-1}(n-t+q)^{3}\right] \end{aligned}  $$

28 $$ = \frac{q^{2}}{2n^{3}}\left(1+2nq-q^{2}\right),  $$

which yields a simple form for *C*_2_ given by

29 $$ C_{2}(n,q,w) = \frac{(n-q+1)(n-q)(n-q-1)}{n^{3}}.  $$

We finally note that it is possible to derive a closed form expression for *C*_2_ in the case of exponential weights that were previously considered for the variance estimator; however, the expression is quite lengthy and we omit it here but it is available upon request. Finally, we turn to a simulation in order to understand for what parameter pairs (*n,q*) the effects of the unbiased skewness estimator are most significant.

## Empirical studies and results

We now develop a simulation to compare the relative error of the uniformly weighted unbiased skewness estimator $\hat {\gamma }_{y}$ and the standard unbiased sample skewness estimator which may be found in Zwillinger and Kokoska (2000). We first construct a dataset of end of day simple returns calculated from closing prices for the SPY exchange traded fund from January 1, 2012, to December 31, 2016. This was achieved using Bloomberg’s Python API and an associated wrapper package named tia. We downloaded historical end of day closing prices identified with Bloomberg’s PX_LAST field that are both split and dividend adjusted. This time series was fully populated with data, and hence, there was no need to fill in missing values.

Next, we fit a model distribution to this data in order to establish a framework to test the weighted unbiased skewness and kurtosis estimators in a setting where the true values of these statistics are known which closely approximates actual market data. To this end, we let *X* denote a skew normal distribution whose probability density function is given by

30 $$ p(x;a,b,c) = \frac{2}{b}\phi\left(\frac{x-a}{b}\right)\Phi\left(c\frac{x-a}{b}\right),  $$

where here *ϕ* and *Φ* are the probability and cumulative distribution functions of a standard normal random variable and *b*>0. The skew normal distribution has mean *μ* and variance *σ*^2^ defined by

31 $$ {\begin{aligned} \mu &= a+bd\sqrt{\frac{2}{\pi}}, \\ \sigma^{2} &= b^{2}\left(1-\frac{2d^{2}}{\pi}\right),\quad \text{where}\quad d = \frac{c}{\sqrt{1+c^{2}}}. \end{aligned}}  $$

The normalized skewness *γ* of this distribution is given by

32 $$ \gamma = \frac{\text{Skew}(X)}{\text{Var}(X)^{3/2}}=\frac{4-\pi}{2}\frac{(d\sqrt{2/\pi})^{3}}{(1-2d^{2}/\pi)^{3/2}}.  $$

We fit this distribution to the SPY daily return data using maximum likelihood estimation. Let the likelihood function of this model be denoted by $\mathcal {L}$. We find the model parameters by directly maximizing the log-likelihood function

33 $$ \begin{aligned} \hat{a},\hat{b},\hat{c} &= \underset{a,b,c}{\text{argmax}}\ln\mathcal{L} = n\ln\left(\frac{2}{b}\right)\\&\quad+\sum_{t=1}^{n}\ln\phi\left(\frac{x_{t}-a}{b}\right) + \Phi\left(c\frac{x_{t}-a}{b}\right) \end{aligned}  $$

using a BFGS optimizer developed in Fletcher (1987) and determine that the best fit parameters are given by $\hat {a}=0.00640$, $\hat {b}=0.01006$, and $\hat {c}=-1.1029$, which have associated mean, variance, and normalized skewness given by 4.528×10^−4^, 6.5789×10^−5^, and − 0.1689, respectively. Returns will be drawn from this distribution in the Monte Carlo simulation considered below. Specifically, this simulation consists of sampling *n* values from the MLE distribution, computing the *q*-period overlapping returns of these time series, then calculating the normalized unbiased sample skewness $\tilde {\gamma }_{s}$ (see Zwillinger and Kokoska (2000)) and the normalized unbiased skewness $\tilde {\gamma }_{y}$ with uniform weights given by $\tilde {\gamma }_{y}= \hat {\gamma }_{y}/\hat {\sigma }_{y}^{3}$. We then compute the relative error $|\tilde {\gamma }_{s}-\tilde {\gamma }_{y}|/\tilde {\gamma }_{y}$, between the different estimators for each simulation, repeat the above process 10,000 times, and display the mean percentage errors in Table 1 for distinct (*n,q*) pairs.

**Table 1:** Mean relative percentage errors between the normalized unbiased sample skewness $\tilde {\gamma }_{s}$ and the normalized unbiased skewness $\tilde {\gamma }_{y}$ for varying sample sizes *n*=32,…,16384, and overlapping return periods *q*=2,…,128

*n*/*q*	2	4	8	16	32	64	128
32	14.24	33.14	68.01	*	*	*	*
64	7.08	16.55	35.36	69.79	*	*	*
128	3.53	8.25	17.71	36.47	70.67	*	*
256	1.76	4.11	8.83	18.29	37.03	71.12	*
512	0.88	2.05	4.41	9.12	18.58	37.31	71.34
1024	0.44	1.03	2.20	4.55	9.27	18.72	37.45
16384	0.03	0.06	0.14	0.28	0.58	1.16	2.34

We omit cases where the number of overlapping returns *n*−*q*≤*q*, and first note that as the sample size increases, the error between the two estimators decreases for any fixed *q* value. However, when *q*/*n* is relatively large, say greater than 5%, then there are significant differences between the two estimators.

Next, we explore several weighting schemes described in Andrews (1991) which are widely used for covariance matrix estimation in the presence of heteroskedasticity and autocorrelation. Specifically, we consider weights constructed by Bartlett (Oppenheim et al. 1999), Parzen (White 1980), Tukey-Hamming (Blackman and Tukey 1958), and the Quadratic Spectral weights of Priestley (1962) and Epanechnikov (1969). These weights are defined in terms of a kernel function *k*(·) and are given by *w*_*t*_=*k*(*bt*/*T*) where *T* is a bandwidth parameter and *b* is a scaling constant in Zeileis (2004) and Zwillinger (2000). There are many references that study the problem of optimal bandwidth selection c.f. (Lazarus et al. 2017; Newey and West 1994; Stock and Watson 2011; Wooldridge 2006); however, we are interested in constructing reasonable weighting schemes to place importance on more recent over prior data. We found that setting the bandwidth to the sample size and *b*=1.2 achieves this aim.

**Fig. 1:**
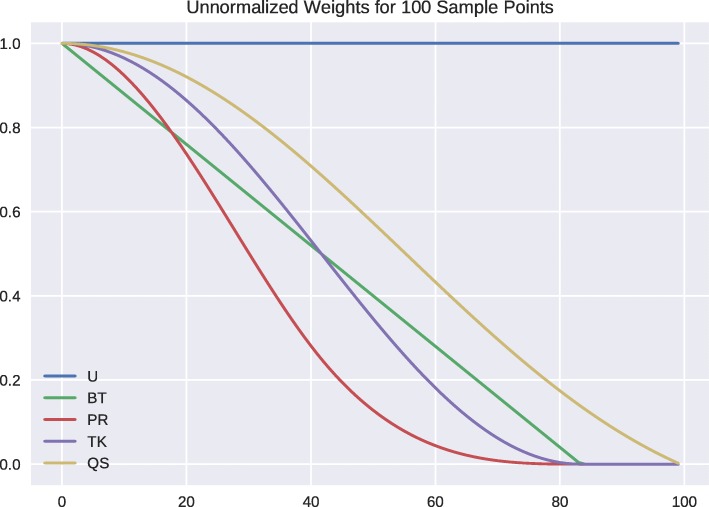
Plot of the unnormalized HAC kernel functions *k*_*BT*_, *k*_*PR*_, *k*_*TK*_, *k*_*QS*_ and the uniform kernel

In Fig. 1, we plot the kernel functions associated with these weighting schemes given in Andrews (1991) which are defined by

34 $$ k_{BT}(x) = \left\{ \begin{array}{ll} 1 - |x| & |x|\leq 1 \\ 0 & |x| > 1, \\ \end{array}\right.  $$

35 $$ k_{PR}(x) = \left\{ \begin{array}{ll} 1 - 6x^{2} + 6|x|^{3} & |x|\leq 1/2 \\ 2(1-|x|)^{3} & 1/2 \leq |x| \leq 1 \\ 0 & |x| > 1, \\ \end{array}\right.  $$

36 $$ k_{TH}(x) = \left\{ \begin{array}{ll} (1+\cos(\pi x))/2 & |x|\leq 1 \\ 0 & |x| > 1, \\ \end{array}\right.  $$

37 $$ k_{QS}(x) = \frac{25}{12\pi^{2}x^{2}}\left(\frac{\sin(6\pi x/5)}{6\pi x/5} - \cos(6\pi x/5)\right).  $$

Note that when using weighted estimators, one effectively reduces the original sample size. For example, in the extreme case of binary zero or one valued weights, only the weights with value one contribute to the estimator which reduces the sample size to the percentage of one valued weights. In this example, we can find the percentage sample size reduction by approximating the area under each weight curve. In reference to the uniform weights which we take to have normalized area of 1, the HAC weights effectively reduce the sample size by PR: 31%, BT,TK: 41%, and QS: 54% so that uniform weights have approximately two to three times the sample size of these weighting schemes.

Next, we examine how estimation of the unbiased weighted standard deviation and skewness estimators varies as a function of the overlapping return period *q*, sample size *n*, and weighting scheme using the SPY dataset previously described. We consider overlapping return periods of 5, 21, and 63 sample points which correspond to weekly, monthly, and quarterly aggregation windows for our example daily return data. Next, we truncate the sample size to 256, 512, and 1024 data points which roughly correspond to 1, 2, and 5-year time periods, using a trailing truncation window on the SPY returns.

Estimation results are presented in Table 2 where overlapping return standard deviations are displayed as percentages. We first note that estimates for uniform weights tend to be outliers as they include two to three times the effective sample size as the other weighting schemes. Next, note that overall standard deviation values are greatest in the *n*=512 case, slightly lower for *n*=256, and considerably lower for *n*=1024. This is due to the historical volatility of the S&P 500 being relatively high during the mid 2015 to early 2016 time period and lower in prior years over the five year window being considered. One may also note that the overlapping return distribution becomes increasingly negatively skewed as a result.

**Table 2:** Comparison of unbiased overlapping return standard deviation and skewness estimators as a function of weighting scheme, sample size *n*, and overlapping return period *q*

	*n*=256			*n*=512			*n*=1024		
	*q*=5	*q*=21	*q*=63	*q*=5	*q*=21	*q*=63	*q*=5	*q*=21	*q*=63
Std(%)									
*U*	1.764	3.146	4.086	1.885	3.459	5.225	1.729	3.130	4.387
*BT*	2.167	3.810	4.359	1.999	3.519	5.330	1.611	2.847	3.845
*PR*	2.409	4.321	4.679	1.894	3.196	4.963	1.512	2.634	3.260
*TK*	2.215	3.892	4.388	1.997	3.480	5.328	1.556	2.730	3.501
*QS*	2.038	3.561	4.246	2.001	3.555	5.380	1.648	2.930	3.978
Skewness									
*U*	− 0.489	− 0.296	0.356	− 1.038	− 0.356	− 0.467	− 0.858	− 0.400	− 0.866
*BT*	− 0.612	− 0.525	0.299	− 1.200	− 0.310	− 0.343	− 0.748	− 0.401	− 0.985
*PR*	− 0.582	− 0.752	0.294	− 1.355	− 0.387	− 0.668	− 0.508	− 0.335	− 0.488
*TK*	− 0.616	− 0.623	0.334	− 1.280	− 0.308	− 0.316	− 0.610	− 0.328	− 0.801
*QS*	− 0.601	− 0.446	0.318	− 1.184	− 0.298	− 0.318	− 0.791	− 0.401	− 0.986

Here, we use the following abbreviations for different kernel based weighting schemes: *U* uniform, *BT* Bartlett, *PR* Parzen, *TK* Tukey-Hanning, and *QS* Quadratic Spectral

**Fig. 2:**
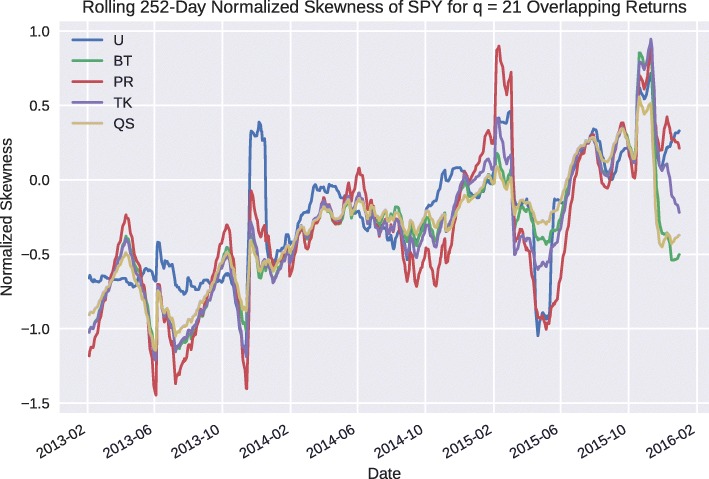
Rolling *n*=252 day overlapping return unbiased skewness for HAC weighting schemes and a *n*=126 lookback period for uniform weights with a *q*=21 overlapping period for SPY daily returns

Finally, we compare how skewness estimation varies over time for different weighting schemes. In particular, we consider a 4-year window from 1/1/2012 to 1/1/2016 and estimate the unbiased skewness using a 252-day lookback period for each of the HAC estimators and a 126-day lookback period for the uniformly weighted estimator which is selected to ensure that the sample sizes are on par with one another. This procedure is carried out on a rolling basis, and results are plotted in Fig. 2.

We note that the general forms of the time series in Fig. 2 tend to be similar for the majority of dates displayed. The HAC weighted estimators are more reactive than the uniform estimator and do not exhibit single day jumps that are as large in magnitude as the HAC uniform estimator.

## Discussion and conclusions

In summary, we have derived closed form expressions for weighted unbiased variance and skewness estimators. We also developed simplified expressions for these estimators in the case of exponential weights for the variance estimator and uniform weights for both estimators. The differences between the standard unbiased sample skewness and new normalized unbiased skewness estimators were demonstrated to be significant in the case of skewness estimation for SPY end of day return data for HAC weighting schemes.

We note that as in Bod et al. (2002) and Lo and MacKinlay (1988), we assume returns satisfy the random walk version of the martingale hypothesis which generally does not hold for financial time series. An interesting future application of the skewness estimator would be to develop a hypothesis test for this assumption which may compliment the results in Lo and MacKinlay (1988). For additional future work, it would be of interest to consider as in Kluitman and Franses (2002) analogues of the weighted unbiased variance and skewness estimators under the assumption that the return process satisfies an AR(1), MA(1) or more general time series model. This will require repeating the above derivations and retaining terms of the form $\mathbb {E}\left [r_{t}r_{s}\right ]$ and $\mathbb {E}\left [r_{t}r_{s}r_{k}\right ]$ for *t*≠*s*≠*k* which are no longer trivial but depend on the underlying model one assumes for the return process. Then, one could fit such models to market data and compare values of the two estimators. We anticipate the estimator will strongly depend on the sign of the AR(1) lag parameter and the white noise parameter of the MA(1) model as shown in Kluitman and Franses (2002) but leave this for a future study.
